# Association of plain water intake with risk of all-cause and cause-specific mortality in individuals with non-alcoholic fatty liver disease or metabolic dysfunction-associated steatotic liver disease

**DOI:** 10.3389/fnut.2024.1478194

**Published:** 2024-10-30

**Authors:** Na Zhao, Yun He, Yuan Li, Ning Zhang, Yan Wang

**Affiliations:** Department of Gastroenterology and Hepatology, The Second Hospital of Hebei Medical University, Shijiazhuang, China

**Keywords:** plain water, non-alcoholic fatty liver disease, metabolic dysfunction-associated steatotic liver disease, mortality, NHANES

## Abstract

Non-alcoholic fatty liver disease (NAFLD) or metabolic dysfunction-associated steatotic liver disease (MASLD)-related mortality have increased dramatically in past decades. Our study aims to investigate the association between plain water and this prevalent metabolic disease, as water plays a crucial role in regulating metabolic processes. A total of 3,543/3,428 individuals with NAFLD/MASLD were included in this study from National Health and Nutrition Examination Survey (NHANES). Daily plain water intake was recorded, and mortality status was tracked until December 31, 2019. Multivariate Cox regression models and restricted cubic spline (RCS) regression models were used to assess the association between plain water intake and long-term all-cause as well as cause-specific mortality among participants with NAFLD/MASLD. Furthermore, we investigated the relationship between substituting other beverages with plain water intake and the risk of mortality. The multivariate Cox regression analyses revealed a significant association between higher plain water intake and lower all-cause mortality, cerebrovascular diseases mortality, and cancer mortality in both NAFLD or MASLD patients. Dose-response analyses revealed a non-linear trend between plain water intake and mortality among NAFLD/MASLD patients. Additionally, replacing sugar or artificial beverages with plain water was linked to reduced all-cause mortality, cerebrovascular diseases mortality, and cancer mortality in patients with NAFLD/MASLD. Higher plain water intake is independently linked to lower risk of all-cause, cerebrovascular diseases mortality, and cancer mortality in NAFLD/MASLD patients. Increasing plain water intake may be an effective way for these patients to reduce their risk of mortality.

## Introduction

The global prevalence of non-alcoholic fatty liver disease (NAFLD) has surpassed previous estimates and is projected to continue rising, affecting over 30% of the adult population worldwide (1, 2). In the United States, NAFLD affects nearly 100 million individuals and stands as one of the most prevalent chronic liver diseases (3). The presence of NAFLD is a well-known risk factor for liver-related endpoints, including end-stage liver disease and hepatocellular carcinoma (4–6). It also increases the risk of overall and specific-cause mortality, with cardiovascular-related mortality being the primary cause among individuals with NAFLD (7, 8). However, following the Delphi consensus process, it was proposed to use “steatotic liver disease (SLD)” as a substitute for “fatty liver disease.” Additionally, it was recommended to use “metabolic dysfunction-associated steatotic liver disease (MASLD)” instead of “NAFLD” (9).

The cornerstone of NAFLD/MASLD treatment remains the regulation of dietary nutrients, which has the potential to reverse the presence of steatosis and non-advanced fibrosis (6–8). Water, an essential nutrient, is believed to be related to a variety of health outcomes. It plays a crucial role in regulating metabolic processes, contributing to the maintenance of homeostasis and overall physiological function (10, 11). Available evidence indicates that metabolic disorders are the primary risk factors for the development of NAFLD/MASLD (12). Metabolic disorders are closely associated with a decrease in energy expenditure relative to intake, as well as abnormal lipid metabolism (13). Increasing plain water intake and substituting caloric beverages with plain water have been proven effective methods for reducing total energy intake and increasing lipid oxidation and energy expenditure (14–16). Additionally, recent finding suggests that increased consumption of plain water may be linked to a lower risk of newly diagnosed NAFLD, indicating that plain water plays a role in regulating metabolic disorders and may have a protective effect on NAFLD (17). However, it remains uncertain whether the role of plain water intake in patients diagnosed with NAFLD/MASLD will affect their subsequent risk of mortality.

Therefore, our study aimed to investigate the association between plain water intake and the all-cause mortality risk in a large cohort of adult NAFLD/MASLD patients using data from the US NHANES spanning from 1988 to 2018. Additionally, we also aimed to detect the dose-response relationship between the consumption of plain water and the risk of mortality among patients with NAFLD/MASLD. Finally, our study investigated the effect of substituting beverage intake with plain water consumption and its association with all-cause and cause-specific mortality.

## Methods

### Study design and participants

The NHANES program, carried out by the NCHS of the Centers for Disease Control and Prevention, is designed to evaluate the health and nutritional status of individuals in the United States. It utilizes a complex multistage probability sampling design to choose a representative sample of the civilian non-institutionalized household population. Data were collected from NHANES III (1988–1994) and subsequent NHANES surveys from 1999 to 2018. NHANES III covered a span of 6 years, with public data available for each 3-year phase as well as the combined 6-year period. Starting from 1999, NHANES became an annual survey, with public data including combined information for every 2 years. During each survey, sampled individuals undergo a household interview and receive a comprehensive physical examination in a specially equipped Mobile Examination Center (MEC). Anthropometric, dietary, and laboratory data are gathered during the MEC examination. The response rates for the MEC-examined sample in each NHANES used were at 70% (18). The protocol for NHANES was approved by the NCHS Research Ethics Review Board, and written consent was obtained from each participant.

### Definitions of SLD, NAFLD and MASLD

The presence of hepatic steatosis was identified using ultrasonic examinations. Individuals with hepatic steatosis (including mild, moderate, and severe cases) were diagnosed with SLD. NAFLD was defined as the presence of SLD without excessive alcohol intake or positive hepatitis viral infection (19). MASLD was defined as having SLD plus at least one of the following cardiometabolic adult criteria without other causes of hepatic steatosis or excessive alcohol consumption (≥30 g/d for males and ≥20 g/d for females) (9). The cardiometabolic adult criteria included: (1) body mass index (BMI) ≥25 kg/m^2^ (23 kg/m^2^ for Asia) or waist circumference (WC) >94 cm for males and >80 cm for females; (2) fast blood glucose (FBG) ≥5.6 mmol/L or 2-h post-load glucose levels ≥7.8 mmol/L or glycated hemoglobin (HbA1c) ≥5.7% or type 2 diabetes or treatment for type 2 diabetes; (3) systolic blood pressure (SBP)/diastolic blood pressure (DBP) ≥130/85 mmHg or specific anti-hypertensive drug treatment; (4) triglycerides (TG) ≥1.70 mmol/L or lipid lowering treatment; and (5) plasma high-density lipoprotein cholesterol (HDL-C) ≤1.0 mmol/L for males and ≤1.3 mmol/L for females or lipid lowering treatment. Non-invasive liver fibrosis assessment included the Fibrosis Score (NFS) and the fibrosis-4 index (FIB-4), which were utilized for diagnosing advanced fibrosis. The cut-off values for NFS and FIB-4 were −1.455 and 1.3, respectively (20).

### Exposure assessment

The study examined various factors such as the consumption of plain water and the moisture content of foods and drinks. Data on daily water intake was collected after participants completed a 24-h dietary recall, including tap water, spring water, noncarbonated bottled water, and other sources. The automated multiple pass method was used to collect beverage and food water intake data for large-scale national surveys. Total water intake was calculated from plain water, beverages, and food. Trained staff collected and reviewed the data on water intake before entering it into an electronic system.

### Outcome assessment

We analyzed the mortality data from the 2019 public-linked mortality files provided by the National Center for Health Statistics (NCHS). The NCHS primarily identified mortality status through probabilistic matching to the National Death Index (NDI), with additional sources of mortality-status information including the Social Security Administration, the Centers for Medicare and Medicaid Services, and death certificates (21). The mortality data from the public domain were modified to ensure anonymity while maintaining the integrity of vital-status information. The public domain mortality data were adjusted to protect anonymity while preserving vital-status information. The linked files contained detailed information on various causes of mortality in the US population based on International Classification of Diseases-10 (ICD-10) cause-of-death codes. For NHANESs 1999–2014, a total of 10 primary causes of mortality were documented, including “Heart diseases,” “Cancer (malignant neoplasms),” “Chronic lower respiratory diseases,” “Unintentional injuries,” “Cerebrovascular diseases,” “Alzheimer’s disease,” “Diabetes,” “Pneumonia and influenza,” “Kidney diseases” and “Other unspecified causes.” In contrast, NHANESs 2015–2018 recorded only three leading causes of death in the linked mortality files: “Heart diseases,” “Cancer,” and “Other unspecified causes.” Therefore, we used available data on these common leading causes of death: “All Causes,” “Cerebrovascular diseases,” “Heart Diseases” and “Malignant Neoplasms” as outcomes of interest in our study covering NHANESs 1999–2018. All-cause mortality was defined as death resulting from any cause. However, specific dates of death were not disclosed; instead, person-months of follow-up starting from the date of the MEC visit or household interview were provided.

### Covariates

Data on potential associations between our exposures and the outcome were available for each survey. The variables included sex, race-ethnicity (Mexican American, non-Hispanic White, non-Hispanic Black, and other Race), level of education (less than high school, high school diploma or equivalent, and above high school), marital status (married/living with partner and widowed/divorced/separated), family income (under $20,000 and $20,000 and over), smoking habits as indicated by serum cotinine concentration, and sedentary lifestyle. In addition, measurements were taken for BMI and WC; levels of TG, HDL, HbA1c, and FBG; SBP and DBP. Additionally obtained was information on total energy intake and moisture in food from 24-h dietary recall.

### Statistical methods

The baseline characteristics were analyzed and presented based on the daily intake of plain water. Continuous variables were expressed as means with a 95% confidence interval (CI), while categorical variables were represented as frequencies and percentages.

The follow-up person-time was calculated from the time of the MEC examination (conducted between 1988 and 1994, and 1999 to 2018) until the most recent known date of the person’s survival. Cox proportional hazard regression models were employed to calculate the hazard ratios (HRs) and 95% CIs in the analyses. The backward stepwise regression method was utilized to identify statistically significant covariates in both univariable and multivariable models, which were subsequently included in the final models for analysis. Final models, adjusted for potential confounders, were used to assess the associations between plain water intake and mortality from all-cause, cerebrovascular diseases, heart disease, cancer, and other causes among patients with NAFLD/MASLD. Other factors were included in the final model after screening covariates using the backward stepwise regression method. We utilized RCS functions to examine the nonlinear relationship between plain water intake and all-cause, heart disease, cancer, and other cause mortality (22, 23). The final cox models and confounders were employed in this analysis to explore the nonlinear association. In line with previous research recommendations, we selected the median plain water intake as the reference value for all analyses of nonlinear association. The fit of nonlinear curves was optimized when incorporating three knots into the models; this approach prevented accuracy reduction due to over-fitting (24). For substitution analyses, isointake substitution models were constructed, incorporating both plain water intake and total beverage intake (25, 26). The coefficients of these models represented the anticipated effects of replacing 100 mL of beverage with 100 mL of plain water. The coefficients of this model indicated the changes in outcomes resulting from substituting beverages with plain water. Covariates were adjusted for in all models.

All analyses were conducted using R software version 4.3.1, and statistical significance was determined at a two-tailed *p*-value of less than 0.05.

## Results

### Study participants

**Figure 1:**
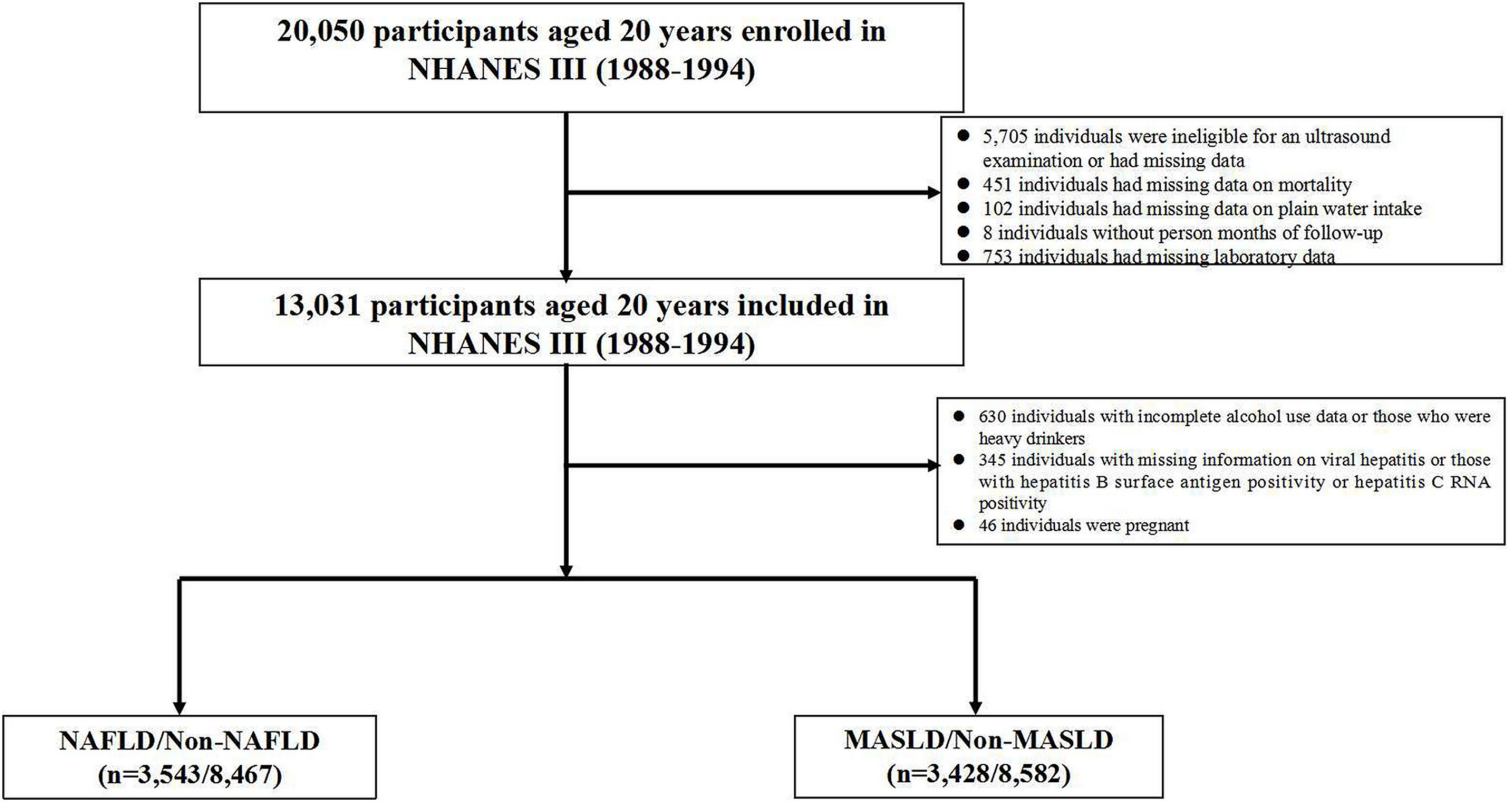
The flow chart for the selection of NAFLD and MASLD patients. NAFLD, non-alcoholic fatty liver disease; MASLD, metabolic dysfunction-associated steatotic liver disease.

The NHANES III enrolled a total of 20,050 participants who were aged 20 years and older. We excluded 5,705 individuals who were ineligible for an ultrasound examination or had missing data, as well as 436 individuals who were ineligible for mortality follow-up. In addition, detailed information regarding the underlying causes of death was not recorded for 15 participants. We also excluded 102 participants with missing data on daily plain water intake and 8 participants without person months of follow-up from the MEC date. Furthermore, we excluded 753 individuals with missing laboratory data. A total of 630 heavy drinkers and individuals who had missing data on alcohol consumption per day. In addition to the above exclusions, we removed 345 participants with missing information on viral hepatitis or those with hepatitis B surface antigen positive or hepatitis C RNA positive. Furthermore, we excluded 46 pregnant participants. Finally, a total of 12,010 individuals with completed ultrasonography and laboratory data were included from the NHANES III database. NAFLD was diagnosed in 3,543 out of 12,010 participants (29.5%), while MASLD was diagnosed in 3,428 out of 12,010 participants (28.5%) within the overall population. Figure 1 illustrates the flow chart for selection of NAFLD and MASLD patients.

### Descriptive analyses

Table 1 presents the baseline characteristics of the study population. The study included a total of 3,543/3,428 patients with NAFLD/MASLD. The mean age was 45.9 years (95% CI = 45.4–46.4) and 45.8 years (95% CI = 45.3–46.3) among NAFLD/MASLD patients. About 29.5% or 28.5% participants were NAFLD/MASLD patients. Nearly half of the subjects were non-Hispanic White, and more than half were married or living with a partner. Approximately half of the individuals had a family income per year less than $20,000, and more than half had received a high level education. At baseline, 871 (24.6%), 815 (23.0%), 964 (27.2%), and 893 (25.2%) NAFLD patients, as well as 771 (22.5%), 816 (23.8%), 973 (28.4%), and 868 (25.3%) MASLD patients were distributed into four groups based on their plain water consumption, with approximately one in three of the individuals being diagnosed with advanced fibrosis using non-invasive methods. During a median follow-up period of 12.8 years, a total of 1,466/1,508 deaths were identified among patients with NAFLD/MASLD. Using NAFLD criteria, 391 individuals died from cerebrovascular diseases, 325 from heart diseases, and 204 from cancer. When MASLD criteria was applied, 398 deaths were attributed to cerebrovascular diseases, 330 to heart diseases, and 217 to cancer.

**Table 1:** Baseline characteristics stratified by plain water intake in the U.S. adult population with NAFLD/MASLD, NHANES III (1988–1994).

Characteristics	Patients with NAFLD (*n* = 3,543)				Patients with MASLD (*n* = 3,428)			
	Quantile 1	Quantile 2	Quantile 3	Quantile 4	Quantile 1	Quantile 2	Quantile 3	Quantile 4
Number of patients	871 (24.6%)	815 (23.0%)	964 (27.2%)	893 (25.2%)	771 (22.5%)	816 (23.8%)	973 (28.4%)	868 (25.3%)
Age [years; mean (95% CI)]	46.0 (45.2–46.7)	46.2 (45.5–47.0)	46.4 (45.8–47.0)	45.5 (44.9–46.2)	45.5 (44.7–46.2)	46.0 (45.3–46.8)	46.5 (45.9–47.2)	44.8 (44.2–45.6)
Sex, male, *n* (%)	482 (26.1%)	421 (22.8%)	476 (25.7%)	469 (25.4%)	343 (20.9%)	394 (24.0%)	492 (29.9%)	412 (25.1%)
Race-ethnicity								
Mexican American	229 (26.3%)	247 (30.3%)	270 (28.0%)	270 (30.3%)	210 (27.3%)	240 (29.4%)	283 (29.1%)	287 (33.1%)
Non-Hispanic White	440 (50.5%)	362 (44.4%)	446 (46.3%)	363 (40.7%)	397 (51.5%)	354 (43.4%)	499 (51.3%)	330 (38.1%)
Non-Hispanic Black	101 (11.6%)	118 (14.5%)	148 (15.4%)	179 (20.1%)	77 (10.0%)	123 (15.1%)	101 (10.4%)	202 (23.3%)
Other Race	101 (11.6%)	88 (10.8%)	100 (10.4%)	81 (9.07%)	87 (11.3%)	45.899 (12.1%)	90 (9.24%)	49 (5.64%)
Marriage status								
Married/living with partner	570 (65.4%)	530 (65.0%)	632 (65.6%)	577 (64.6%)	513 (66.5%)	519 (63.6%)	627 (64.4%)	530 (61.1%)
Widowed/divorced/separated	301 (34.5%)	285 (35.0%)	332 (34.4%)	316 (35.4%)	258 (33.5%)	297 (36.4%)	346 (35.6%)	338 (38.9%)
Family income								
Under $20,000	373 (42.8%)	372 (45.6%)	463 (48.0%)	439 (49.2%)	334 (43.3%)	371 (45.5%)	459 (47.2%)	381 (43.9%)
$20,000 and over	498 (57.2%)	443 (54.4%)	501 (52.0%)	454 (50.8%)	437 (56.7%)	445 (54.5%)	514 (52.8%)	487 (56.1%)
Educational level								
Less than high school	141 (16.2%)	143 (17.5%)	169 (17.5%)	152 (17.0%)	132 (17.1%)	135 (16.6%)	182 (18.7%)	149 (17.2%)
High school or equivalent	312 (35.8%)	259 (31.8%)	294 (30.5%)	261 (29.2%)	257 (33.3%)	262 (32.1%)	313 (32.2%)	256 (29.5%)
Above high school	418 (47.9%)	413 (50.7%)	501 (52.0%)	480 (53.7%)	382 (49.5%)	419 (51.3%)	478 (49.1%)	463 (53.3%)
Sedentary lifestyle, *n* (%)	214 (24.0%)	187 (21.0%)	262 (29.5%)	225 (25.3%)	173 (20.0%)	194 (22.4%)	276 (32.0%)	220 (25.5%)
Serum cotinine [ng/mL; mean (95% CI)]	44.3 (39.4–49.1)	39.1 (34.8–43.4)	46.5 (41.4–51.7)	44.7 (39.8–49.7)	39.3 (34.9–43.6)	44.4 (39.5–49.3)	46.7 (41.6–51.9)	44.8 (39.9–49.8)
BMI [kg/m^2^; mean (95% CI)]	32.0 (31.7–32.3)	31.7 (31.4–32.0)	31.8 (31.5–32.0)	32.7 (32.4–33.0)	31.9 (31.6–32.3)	32.1 (31.8–32.5)	31.6 (31.3–32.0)	32.3 (32.0–32.6)
WC [cm; mean (95% CI)]	108.2 (107.6–108.8)	106.7 (106.1–107.3)	106.9 (106.4–107.5)	108.6 (108.0–109.2)	105.6 (105.0–106.2)	107.1 (106.5–107.7)	106.4 (106.0–107.0)	108.1 (107.5–108.7)
SBP [mmHg; mean (95% CI)]	129.1 (128.3–129.9)	131.4 (130.6–132.3)	131.8 (131.0–132.5)	130.5 (129.7–131.2)	131.0 (130.2–131.9)	129.0 (128.2–129.7)	131.4 (130.6–132.1)	130.2 (129.4–131.0)
DBP [mmHg; mean (95% CI)]	69.9 (69.3–70.6)	70.3 (69.5–71.1)	71.2 (70.5–71.9)	72.7 (72.0–73.5)	70.0 (69.2–70.8)	69.4 (69.0–70.2)	71.0 (70.3–71.6)	72.5 (71.8–73.4)
TG [mg/dL; mean (95% CI)]	179.7 (174.3–185.1)	176.2 (171.2–181.1)	186.9 (181.1–192.6)	190.7 (184.9–196.5)	176.0 (171.0–180.9)	178.2 (173.0–183.6)	186.5 (180.6–192.2)	190.2 (184.4–196.1)
HDL [mg/dL; mean (95% CI)]	45.9 (45.4–46.5)	46.7 (46.1–47.2)	46.3 (45.8–46.9)	45.6 (45.0–46.1)	45.7 (45.1–46.3)	45.4 (45.0–46.0)	45.3 (44.7–45.9)	44.4 (43.8–45.1)
HbA1c [%; mean (95% CI)]	6.02 (5.96–6.07)	6.07 (6.01–6.13)	6.12 (6.07–6.18)	6.06 (5.99–6.13)	6.05 (5.98–6.12)	5.99 (5.92–6.05)	6.10 (6.05–6.16)	6.20 (6.13–6.27)
FBG [mg/dL; mean (95% CI)]	125.3 (123.4–127.3)	128.2 (126.0–130.4)	131.5 (129.4–133.7)	138.8 (135.9–141.7)	127.1 (125.0–129.0)	124.3 (122.4–126.3)	130.0 (128.0–132.2)	138.6 (135.7–141.5)
Plain water intake (g/day)	145.0 (138.8–151.2)	650.9 (644.4–657.5)	1247.3 (1237.6–1257.1)	2786.5 (2732.6–2840.4)	142.0 (136.8–149.5)	655.0 (640.4–653.5)	1237.1 (1227.7–1247.0)	2788.8 (2734.9–2842.8)
Moisture in food (g/day)	384.1 (272.2, 472.2)	735.2 (644.3, 831.1)	1224.1 (1066.2, 1410.2)	2221.1 (1875.4, 2709.2)	354.2 (256.3, 440.3)	704.4 (612.2, 802.5)	1201.1 (1044.1, 1385.2)	2570.6 (2504.4–2635.7)
Beverages (g/day)	473.7 (342.7, 576.9)	837.5 (751.6, 927.5)	1230.4 (1122.4, 1358.5)	1971.2 (1712.1, 2375.2)	568.2 (410.3, 684.2)	1013.1 (921.1, 1126.2)	1431.4 (1309.4, 1573.5)	2201.0 (1942.2, 2601.2)
Total energy intake (kal)	2037.9 (2019.5–2056.5)	1900.2 (1881.3–1918.7)	2001.1 (1982.2–1964.5)	1957.7 (1939.3–1976.3)	2011.1 (1992.7–2029.7)	1988.0 (1969.6–206.6)	1959.5 (1941.1–1978.1)	1968.2 (1949.8–1986.8)
Advanced fibrosis (NFS >−1.455)	282 (32.3%)	288 (35.3%)	279 (28.9%)	273 (30.6%)	281 (32.2%)	280 (34.3%)	299 (31.0%)	297 (33.2%)
Advanced fibrosis (FIB-4 >1.3)	278 (36.0%)	289 (35.4%)	272 (27.9%)	270 (31.1%)	280 (36.3%)	277 (33.9%)	282 (30.0%)	290 (33.4%)
Outcome of mortality								
All-cause	440 (30.0%)	380 (25.9%)	343 (23.3%)	303 (20.7%)	466 (30.9%)	378 (25.1%)	337 (22.3%)	327 (21.7%)
Cerebrovascular diseases	121 (31.0%)	102 (26.1%)	90 (23.0%)	78 (19.9%)	122 (30.7%)	105 (26.4%)	96 (24.1%)	75 (18.8%)
Heart diseases	103 (31.7%)	87 (26.8%)	77 (23.7%)	58 (17.8%)	104 (31.5%)	86 (26.0%)	79 (23.9%)	61 (18.5%)
Cancer	59 (28.9%)	52 (25.5%)	48 (23.5%)	45 (22.1%)	62 (28.6%)	54 (24.9%)	52 (24.0%)	49 (22.6%)
Other causes	161 (29.5%)	141 (25.8%)	129 (23.6%)	115 (21.1%)	172 (30.5%)	144 (25.6%)	138 (24.6%)	109 (19.3%)

NAFLD, non-alcoholic fatty liver disease; MASLD, metabolic dysfunction-associated steatotic liver disease; CI, confidence interval; BMI, body mass index; WC, waist circumference; SBP, systolic blood pressure; DBP, diastolic blood pressure; triglycerides, TG; high-density lipoprotein cholesterol, HDL-C; HbA1c, glycated hemoglobin; FBG, fast blood glucose. Continuous variables were represented as means and 95% CI. Categorical variables were represented as frequencies and percentages.

### Association of plain water intake and mortality of NAFLD/MASLD

The study utilized Cox proportional regression models adjusted for covariates to examine the relationship between plain water intake and the risk of all-cause and cause-specific mortality (Table 2). Patients with NAFLD in the third quartile group of plain water intake, with an average consumption of 1247.3 g/day (95% CI = 1237.6–1257.1), demonstrated a significantly lower risk of all-cause mortality (HR = 0.62, 95% CI = 0.58–0.67), cerebrovascular diseases mortality (HR = 0.54, 95% CI = 0.50–0.61), and cancer mortality (HR = 0.72, 95% CI = 0.65–0.78) compared to those in the first quartile group. Patients with NAFLD in the fourth quartile group of plain water intake with an average consumption of 2786.5 g/day (95% CI = 2732.6–2840.4) exhibited a significantly lower risk of all-cause mortality (HR = 0.60, 95% CI = 0.55–0.65), cerebrovascular diseases mortality (HR = 0.53, 95% CI = 0.50–0.61) and cancer death rate (HR = 0.66, 95% CI = 0.61–0.71) compared to those in the first quartile group. We did not find any association between the consumption of plain water and heart disease-related deaths. The similar results were observed in patients with MASLD (Table 3). However, patients with NAFLD had a lower risk of mortality compared to those with MASLD.

**Table 2:** Association of plain water intake and all-cause and cause-specific mortality in the U.S. adult population with NAFLD, NHANES III (1988–1994) and NHANESs 1999–2018.

Cause of mortality	Plain water intake	HR (univariable)	HR (multivariable)	HR (final)^a^
All-cause mortality				
	Quantile 1	Ref.	Ref.	Ref.
	Quantile 2	0.92 (0.86–1.03)	0.85 (0.80–1.01)	0.85 (0.80–1.01)
	Quantile 3	**0.70 (0.65–0.75)**	**0.62 (0.57–0.67)**	**0.62 (0.58–0.67)**
	Quantile 4	**0.65 (0.60–0.70)**	**0.60 (0.55–0.65)**	**0.60 (0.55–0.65)**
	P-value	<0.001	<0.001	<0.001
Cerebrovascular diseases mortality				
	Quantile 1	Ref.	Ref.	Ref.
	Quantile 2	0.66 (0.44–1.00)	0.69 (0.45–1.04)	0.69 (0.46–1.04)
	Quantile 3	**0.58 (0.54–0.63)**	**0.54 (0.50–0.61)**	**0.54 (0.50–0.61)**
	Quantile 4	**0.50 (0.46–0.54)**	**0.53 (0.50–0.60)**	**0.53 (0.50–0.61)**
	*p*-value	<0.001	<0.001	<0.001
Heart diseases mortality				
	Quantile 1	Ref.	Ref.	Ref.
	Quantile 2	**0.90 (0.85–0.96)**	0.95 (0.90–1.01)	0.95 (0.90–1.01)
	Quantile 3	**0.82 (0.77–0.87)**	**0.78 (0.70–0.91)**	0.87 (0.70–1.02)
	Quantile 4	**0.73 (0.68–0.77)**	**0.72 (0.65–0.79)**	0.82 (0.65–1.02)
	*p*-value	<0.001	<0.001	<0.001
Cancer mortality				
	Quantile 1	Ref.	Ref.	Ref.
	Quantile 2	0.97 (0.92–1.13)	0.90 (0.85–1.15)	0.90 (0.85–1.15)
	Quantile 3	**0.79 (0.74–0.84)**	**0.72 (0.65–0.77)**	**0.72 (0.65–0.78)**
	Quantile 4	**0.70 (0.66–0.75)**	**0.66 (0.61–0.71)**	**0.66 (0.61–0.71)**
	*p*-value	<0.001	<0.001	<0.001
Other causes mortality				
	Quantile 1	Ref.	Ref.	Ref.
	Quantile 2	0.86 (0.78–1.10)	0.84 (0.76–1.11)	0.84 (0.76–1.11)
	Quantile 3	**0.70 (0.63–0.78)**	**0.65 (0.59–0.70)**	**0.65 (0.59–0.70)**
	Quantile 4	**0.56 (0.50–0.63)**	**0.55 (0.49–0.60)**	**0.55 (0.49–0.60)**
	*p*-value	<0.001	<0.001	<0.001

NAFLD, non-alcoholic fatty liver disease; HR, hazard ratio; CI, confidence interval; Ref., reference. Significant values are in bold.

a The backward stepwise regression method was employed to screen for statistically significant covariates in both univariable and multivariable models, which were included in the final models for analysis.

**Table 3:** Association of plain water intake and all-cause and cause-specific mortality in the U.S. adult population with MASLD, NHANES III (1988–1994) and NHANESs 1999–2018.

Cause of mortality	Plain water intake	HR (univariable)	HR (multivariable)	HR (final)^a^
All-cause mortality				
	Quantile 1	Ref.	Ref.	Ref.
	Quantile 2	0.84 (0.71–1.09)	0.80 (0.67–1.05)	0.80 (0.67–1.05)
	Quantile 3	**0.73 (0.62–0.87)**	**0.70 (0.65–0.75)**	**0.70 (0.65–0.76)**
	Quantile 4	**0.72 (0.62–0.83)**	**0.68 (0.63–0.73)**	**0.68 (0.63–0.73)**
	*p*-value	<0.001	<0.001	<0.001
Cerebrovascular diseases mortality				
	Quantile 1	Ref.	Ref.	Ref.
	Quantile 2	0.90 (0.84–1.07)	0.90 (0.83–1.08)	0.90 (0.83–1.08)
	Quantile 3	**0.87 (0.81–0.93)**	**0.86 (0.80–0.92)**	**0.86 (0.80–0.92)**
	Quantile 4	**0.86 (0.80–0.93)**	**0.80 (0.74–0.89)**	**0.80 (0.74–0.89)**
	*p*-value	<0.001	<0.001	<0.001
Heart diseases mortality				
	Quantile 1	Ref.	Ref.	Ref.
	Quantile 2	0.95 (0.88–1.05)	0.93 (0.85–1.07)	0.93 (0.85–1.07)
	Quantile 3	**0.92 (0.85–0.99)**	0.91 (0.75–1.17)	0.91 (0.75–1.17)
	Quantile 4	**0.87 (0.81–0.94)**	0.83 (0.67–1.10)	0.83 (0.67–1.10)
	*p*-value	<0.001	<0.001	<0.001
Cancer mortality				
	Quantile 1	Ref.	Ref.	Ref.
	Quantile 2	0.82 (0.68–1.04)	0.80 (0.65–1.05)	0.80 (0.65–1.05)
	Quantile 3	**0.78 (0.66–0.93)**	**0.77 (0.65–0.92)**	**0.77 (0.65–0.92)**
	Quantile 4	**0.70 (0.60–0.83)**	**0.70 (0.59–0.83)**	**0.70 (0.59–0.83)**
	*p*-value	<0.001	<0.001	<0.001
Other causes mortality				
	Quantile 1	Ref.	Ref.	Ref.
	Quantile 2	0.80 (0.72–1.00)	0.80 (0.72–1.01)	0.80 (0.72–1.01)
	Quantile 3	**0.70 (0.62–0.80)**	**0.71 (0.65–0.78)**	**0.71 (0.65–0.78)**
	Quantile 4	**0.63 (0.55–0.71)**	**0.68 (0.62–0.73)**	**0.68 (0.62–0.73)**
	*p*-value	<0.001	<0.001	<0.001

MASLD, metabolic dysfunction-associated steatotic liver disease; HR, hazard ratio; CI, confidence interval; Ref., reference. Significant values are in bold.

a The backward stepwise regression method was employed to screen for statistically significant covariates in both univariable and multivariable models, which were included in the final models for analysis.

**Figure 2:**
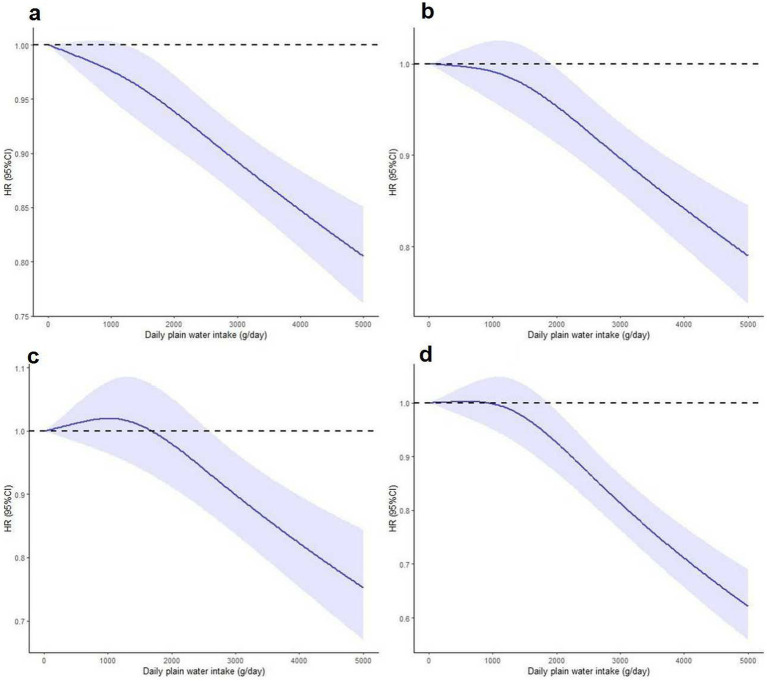
Nonlinear association between plain water intake with risk of all-cause **(a)**, cerebrovascular diseases **(b)**, cancer **(c)** and other cause mortality **(d)** among NAFLD patients. Associations were assessed using multivariable Cox regression models with restricted cubic splines. NAFLD, non-alcoholic fatty liver disease.

**Figure 3:**
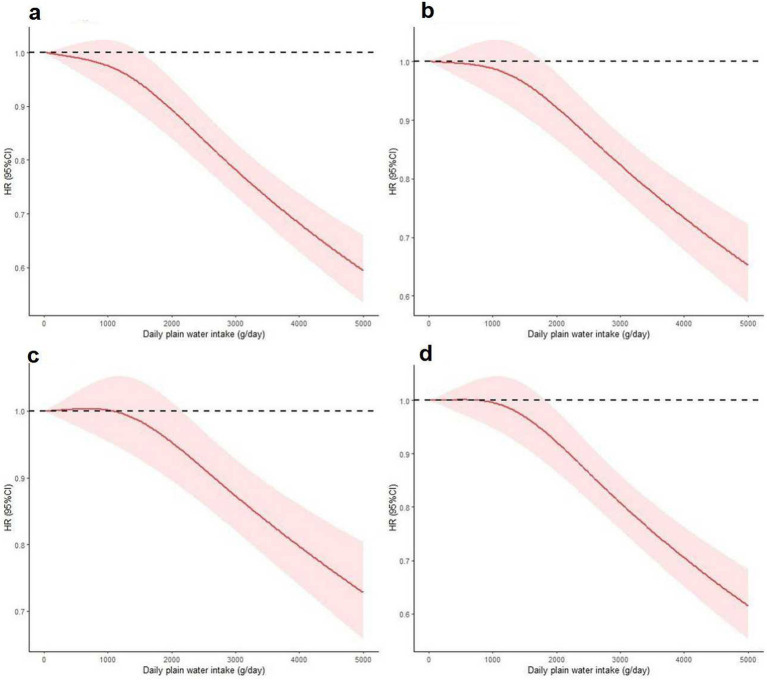
Nonlinear association between plain water intake with risk of all-cause **(a)**, cerebrovascular diseases **(b)**, cancer **(c)** and other cause mortality **(d)** among MASLD patients. Associations were assessed using multivariable Cox regression models with restricted cubic splines. MASLD, metabolic dysfunction-associated steatotic liver disease.

There exists a distinct inverse dose-response relationship between the intake of plain water and both all-cause and cause-specific mortality. Figures 2, 3 demonstrate a statistically significant, non-linear negative association between plain water intake and all-cause specific mortality. This finding holds true with the exception of heart disease-related deaths among NAFLD and MASLD patients, respectively.

### Substitution of beverages intake with plain water consumption

When sugar or artificial beverages were replaced with plain water, a lower all-cause mortality was observed among patients with NAFLD. After adjusting for covariates, a significantly lower risk of cerebrovascular diseases mortality and cancer mortality was found, but not in heart diseases mortality. Similar findings were discovered in patients with MASLD; however, patients with NAFLD had a lower risk of mortality compared to those with MASLD (Table 4).

**Table 4:** Substitution of beverages intake with plain water consumption at a rate of 100 mL per day and its association with all-cause and cause-specific mortality in the U.S. adult population with NAFLD/MASLD, NHANES III (1988–1994) and NHANESs 1999–2018.

NAFLD	Quantile 1	Quantile 2	Quantile 3	Quantile 4
All-cause mortality	Ref.	0.82 (0.68–1.02)	**0.76 (0.69–0.91)**	**0.68 (0.60–0.76)**
Cerebrovascular diseases mortality	Ref.	0.92 (0.85–1.00)	**0.92 (0.86–0.99)**	**0.88 (0.71–0.97)**
Heart diseases mortality	Ref.	0.98 (0.71–1.35)	0.92 (0.67–1.26)	0.91 (0.65–1.28)
Cancer mortality	Ref.	0.86 (0.71–1.03)	**0.76 (0.63–0.92)**	**0.73 (0.62–0.86)**
Other causes mortality	Ref.	0.80 (0.62–1.00)	**0.72 (0.60–0.95)**	**0.62 (0.51–0.80)**

MASLD	Quantile 1	Quantile 2	Quantile 3	Quantile 4
All-cause mortality	Ref.	0.90 (0.84–1.03)	**0.87 (0.81–0.93)**	**0.86 (0.80–0.93)**
Cerebrovascular diseases mortality	Ref.	0.95 (0.80–1.09)	0.93 (0.77–1.05)	**0.88 (0.76–0.99)**
Heart diseases mortality	Ref.	0.99 (0.88–1.54)	0.99 (0.74–1.32)	0.91 (0.67–1.25)
Cancer mortality	Ref.	0.92 (0.85–1.00)	**0.92 (0.85–0.99)**	**0.91 (0.84–0.99)**
Other causes mortality	Ref.	0.84 (0.71–1.00)	**0.73 (0.62–0.87)**	**0.72 (0.64–0.81)**

NAFLD, non-alcoholic fatty liver disease; MASLD, metabolic dysfunction-associated steatotic liver disease; HR, hazard ratio; CI, confidence interval; Ref., reference. Significant values are in bold.

## Discussion

In this study of a cohort of individuals with NAFLD/MASLD, we have observed a potentially inverse association between the plain water consumption and the risk of all-cause and cause-specific mortality. Our findings suggest that higher levels of plain water intake may be independently linked to a reduced risk of mortality from various causes, including cerebrovascular diseases and cancer, in participants with NAFLD/MASLD. The relationship between plain water intake and mortality appears to be non-linear, as demonstrated by the RCS curves for all-cause and cause-specific mortality in these patients. Additionally, our results indicate that substituting other beverages with plain water may be associated with a decreased risk of mortality from various causes. In conclusion, our study suggests that consuming plain water may offer potential benefits for prolonging the lives of individuals with NAFLD/MASLD.

The most recent report on NAFLD-related mortality in the United States, utilizing data from the Centers for Disease Control and Prevention Wide Ranging Online Data for Epidemiologic Research database, revealed a significant increase in NAFLD-related mortality between 1999 and 2022. The age-adjusted mortality rate rose from 0.2 to 1.7 per 100,000 individuals, with an average annual percent change of 10.0% (*p* < 0.001) (27). A substantial body of clinical evidence suggested that NAFLD is not only linked to an increased incidence of liver-related morbidity and mortality, but also to a heightened risk of developing other significant extra-hepatic conditions. These include cerebrovascular diseases, which stands as the predominant cause of death in NAFLD patients, as well as extra-hepatic cancers, type 2 diabetes mellitus, and chronic kidney disease (28). As a result, NAFLD placed a significant health and economic burden on a global scale and often results in reduced quality of life and shortened lifespan. However, available evidence demonstrated a correlation between higher total water intake and reduced mortality risks in the general population. The amount of water intake was negatively associated with all-cause mortality risk (17). Our study reported that higher levels of plain water intake are independently associated with a reduced risk of all-cause mortality, cerebrovascular diseases mortality, and cancer diseases mortality among participants with NAFLD/MASLD. Moreover, a non-linear negative associations between plain water intake and all-cause and cause-specific mortality was also observed in our analyses. Hence, our results indicated that plain water may have potential benefits for prolonging the lives of individuals with NAFLD/MASLD.

Beverages, including sugar-sweetened and artificial, have long been considered one of the most important risk factors for all-cause and cause-specific mortality (29–31). Recent meta-analyses, which pooled 32 articles, concluded that the associations were dose-dependent. They found that a daily increase in sugar-sweetened beverage intake of 250 mL was associated with an increased risk of stroke, cancer, and all-cause mortality (32). Substituting the consumption of beverages with plain water has been demonstrated to be a more effective approach in limiting beverage intake. Furthermore, it has been established that this substitution is associated with a reduced incidence of metabolic diseases and mortality rates (33, 34). Consistently, our study also revealed that replacing the consumption of beverages with plain water at a rate of 100 mL per day was associated with a 32 and 14% lower risk of all-cause mortality among patients with NAFLD or MASLD, respectively. Additionally, this substitution method was also linked to lower mortality specifically related to cerebrovascular diseases and cancer.

The health benefits of increasing plain water intake have been widely recognized by researchers in previous studies. Previous research has suggested that higher consumption of plain water is associated with a reduced risk of various chronic metabolic diseases, such as type 2 diabetes (35), hypertension (36), and obesity (37). This indicates that plain water may play a role in the regulation of metabolic disorders and could potentially have a protective effect against such diseases. Furthermore, additional studies have suggested that increased consumption of plain water (≥4 cups/day) may be linked to a decreased risk of newly diagnosed NAFLD (16). High plain water intake has been shown to stimulate energy and fat metabolism (14, 38), playing a crucial role in the regulation of thermogenesis by the sympathetic nervous system as it relates to weight control, fat oxidation, and anti-obesity properties (39, 40). It has been observed that drinking a large glass of plain water increases sympathetic activity (as indicated by elevated plasma noradrenaline concentrations) and enhances sympathetic neural activity in skeletal muscle. This leads to the concept that consuming plain water may serve as a strategy for promoting thermogenesis and fat oxidation (41, 42). Therefore, these findings provide valuable support for the hypothesis that higher plain water intake is associated with a reduced risk of NAFLD/MASLD mortality. Further research is necessary to validate this hypothesis.

The current research has several notable strengths. Firstly, it was a large-scale study conducted on the general population, with comprehensive and well-examined data, thereby enhancing the statistical power of the findings. Furthermore, adjustments were made for a wide range of potential factors that could influence the results, including sociodemographic variables, blood indicators, lifestyle risk factors, and dietary intake. Lastly, ultrasonic examinations were used as the diagnostic standard for hepatic steatosis in our study, providing higher sensitivity and specificity compared to other methods such as (US-FLI) (43), Framingham steatosis index (FSI) (44), and fatty liver index (FLI) used in previous studies (45). However, there are several limitations that should be noted in this study. Firstly, anthropometric measurements and blood indicators were only collected at baseline. Secondly, due to the nature of this study being observational, it is possible that there were underlying biases such as unaccounted for variables that could not be entirely eliminated. Lastly, the study only included patients from the U.S., so the practical clinical application in diverse racial populations from other countries needs to be further validated.

## Conclusion

In conclusion, our study has demonstrated a significant negative association between higher plain water intake and the risk of all-cause and cause-specific mortality in patients with NAFLD/MASLD. It is imperative that future prospective cohort studies or randomized trials be conducted to elucidate the causality.

## Data Availability

Publicly available datasets were analyzed in this study. This data can be found here: https://www.cdc.gov/nchs/nhanes/index.htm.
